# The Prevalence of Sarcopenia in Crohn's Disease Patients and Its Correlation With Disease Activity and Effect on Prognosis

**DOI:** 10.7759/cureus.83579

**Published:** 2025-05-06

**Authors:** Srijith Kadavanoor, Susruth Krishnadas P, Naufal Peumpalath, Sunil Kumar K, Sithara K Balagopal

**Affiliations:** 1 Department of Gastroenterology, Government Medical College Hospital, Thrissur, IND; 2 Department of Gastroenterology, Government Medical College Hospital, Kozhikode, IND; 3 Department of Radiodiagnosis, Government Medical College Hospital, Kozhikode, IND

**Keywords:** clinical remission, endoscopic disease activity, inflammatory bowel disease, prevalence, sarcopenia

## Abstract

Introduction

Crohn’s disease (CD) is a chronic inflammatory disorder that can involve any part from mouth to anus but predominantly involves the distal small intestine and proximal colon. Sarcopenia is recognized as muscle failure consequent to loss of skeletal muscle strength, function, and mass. Sarcopenia was found to be prevalent in CD patients.

Objectives

The primary objective of our study was to assess the prevalence of sarcopenia in CD patients, its correlation with disease activity, and the effect of sarcopenia on the prognosis of CD. The secondary objectives were to find out whether there is any correlation of sarcopenia with C-reactive protein (CRP), erythrocyte sedimentation rate (ESR), albumin, and hemoglobin levels.

Materials and methods

This was a prospective cohort study done on 70 newly diagnosed CD patients in the Department of Gastroenterology, Government Medical College, Kozhikode, Kerala, India. Sarcopenia was assessed with the L3 skeletal muscle index (L3SMI) using a cross-sectional image at the L3 level from a cross-sectional image using Image J software. The cut-off of L3SMI was < 36.5 cm²/m² and 30.2 cm²/m² for male patients and female patients, respectively. The prevalence of sarcopenia was assessed. Its effect on clinical and endoscopic disease severity as well as blood parameters was assessed, and the outcome was measured by clinical remission, response to therapy, and number of disease flares.

Results

The study included 70 newly diagnosed CD patients above 18 years. Out of 70 patients, 26 were female patients and 44 were male patients. The mean age of the study population was 29.86. Mean L3SMI of the population was 37.35 ± 6.63 cm^2^/m^2^. Prevalence of sarcopenia was 38.6% (n=27). The sarcopenia group had a younger age of disease onset (24.52 ± 8.22). Body mass index (BMI) was lower in the sarcopenia group (15.12 ± 1.33 kg/m^2^). Patients with sarcopenia had higher clinical disease severity according to the Crohn's disease activity index (CDAI) score and higher endoscopic severity according to the simple endoscopic score (SES-CD score). Hemoglobin and albumin were lower, and CRP and ESR were higher in the sarcopenia group. Remission at six-month follow-up was 48.1% in the sarcopenia group and was lower when compared to the group without sarcopenia. The number of disease flares was higher in the sarcopenia group (1.07 ± 1.035).

Conclusion

Sarcopenia is prevalent in CD patients. Patients with sarcopenia had higher clinical and endoscopic disease severity. There is decreased remission and an increased number of flares in patients with sarcopenia. Sarcopenia correlated with hemoglobin, albumin, CRP, and ESR.

## Introduction

Inflammatory bowel disease (IBD) comprises conditions characterized by chronic or relapsing immune activation and inflammation in the gastrointestinal tract. Crohn’s disease (CD) and ulcerative colitis (UC) are the two major forms of IBD. The etiology of IBD is currently unknown but appears to be multifactorial. The current hypothesis is that CD and UC result from overly aggressive T cell-mediated immune responses to specific components of intestinal microbiota in genetically susceptible individuals [1]. CD is a chronic inflammatory disorder that can involve any part from mouth to anus but predominantly involves distal small intestine and proximal colon. One-third of patients with CD have involvement of both the ileum and colon, and one-third have disease limited to the ileum [2,3]. Clinical manifestations of CD vary with the location of the disease, and ileocecal disease can be an insidious disease. Some may present with small bowel obstruction, precipitated by impaction of indigestible food materials. Many years of subclinical inflammation can allow progression to fibrotic stenosis, leading to pain, nausea, and vomiting. Patients with active inflammation present with anorexia, loose stools, and weight loss, and clinical examination reveals evidence of malnutrition. The colonic disease can involve mainly the right colon or can extend to involve most of the colon, and the rectum is not often involved or may be less severely inflamed than other colonic segments [4]. The most typical presenting symptom of the colonic disease is diarrhea, occasionally with the passage of obvious blood. The Crohn’s disease activity index (CDAI) is a numerical calculation derived from the sum of products from a list of eight items and multiplied by weighting factors for each item to define the severity of “disease activity” in patients with CD [5]. The most common endoscopic scoring system in CD is the simple endoscopic score for CD (SES-CD). Endoscopic response is defined as an SES-CD reduction of 50%. Endoscopic healing is defined as a SES-CD <3 and absence of ulceration [6]. 

Sarcopenia was initially described in 1989 by Rosenberg to describe changes in body composition such as age-related loss of muscle mass and function [7]. The operational definition of sarcopenia by the European Working Group on Sarcopenia in Older People (EWGSOP2) is based on three criteria: (i) Low muscle strength; (ii) Low muscle quantity or quality; and (iii) Low physical performance.

Criterion 1 fulfillment is probable sarcopenia, and diagnosis is confirmed by criterion 2. If all three criteria are met, sarcopenia is considered severe. The Strength, Assistance in walking, Rising from a chair, Climbing stairs, Falls history questionnaire (SARC-F) questionnaire is recommended by EWGSOP2; it is a five-item questionnaire that is self-reported by patients as a screen for sarcopenia risk [8].

Muscle strength assessment by grip strength is a simple and inexpensive method. Accurate measurement of grip strength is done by a hand-held dynamometer. Grip strength correlates moderately with strength in other body compartments, so it serves as a reliable surrogate for more complicated measures of arm and leg strength [9,10].

Skeletal muscle quantity is measured by various techniques like appendicular skeletal muscle mass or muscle cross-sectional area of a specific group of muscles. CT images of the third lumbar vertebrae (L3) correlated significantly with whole body muscle. As a result, this imaging method has been used to detect low muscle mass, even in patients with normal or high body weights, and it can also predict prognosis [11,12]. Cross-sectional abdominal CT imaging is often part of the routine assessment of IBD and thus no additional scans are required. Physical performance can be assessed by gait speed. Gait speed is considered a quick, safe, and highly reliable test for sarcopenia [13]. A commonly used gait speed test is called the four-meter usual walking speed test, with speed is measured manually with a stopwatch. A single cut-off speed ≤0.8 m/s is advised by EWGSOP2 as an indicator of severe sarcopenia.

It is seen that a greater degree of malnutrition is seen in patients with CD when compared to UC [14]. This may be due to the impact of CD on the small bowel, which is the main site of absorption, or the persistent nature of CD, as a sequela to fistulation, short bowel due to resections or partial obstructive symptoms secondary to stricturing disease [15]. Chronic inflammation is believed to be the key driver of sarcopenia in IBD. Treatment at present aims to target inflammation with less focus on the prevalence and impact of sarcopenia in IBD. Sarcopenia has been shown to have a clear impact on the quality of life, length of hospital stay, surgical outcomes, and mortality [16]. Sarcopenia is thus an important consideration in the management of IBD, in both prognostication and treatment. Whilst there is evolving research about interventions to overcome sarcopenia in many chronic inflammatory states, such as chronic liver disease and rheumatoid arthritis, the same cannot yet be said for IBD. Further research is required to incorporate recognition of sarcopenia into the standard nutritional assessment and to utilize this to tailor individual management. Considering the relevance of sarcopenia in CD, we intended to study the prevalence of sarcopenia in CD patients, its correlation with disease activity, and the effect of sarcopenia on the prognosis of CD. The secondary objectives were to find out whether there is any correlation between sarcopenia with CRP, ESR, albumin, and hemoglobin levels. 

## Materials and methods

This was a prospective cohort study done in the Department of Gastroenterology at Government Medical College Kozhikode, Kerala, India, for a period of one year from November 2021 to October 2022. This included a recruitment period of six months and a follow-up of six months. 

The sample size was calculated using OpenEpi version 3.1. The sample size was calculated based on a previous study that reported the prevalence of sarcopenia as 43% [17]. With an assumption of the anticipated prevalence of 43%, precision of 12%, and design effect of one, the required sample size was estimated to be 66.

All newly diagnosed CD patients older than 18 years who were diagnosed based on clinical, radiological, and endoscopic evaluation and histological confirmation, who attended an outpatient clinic or were admitted to the Gastroenterology ward at Government Medical College Kozhikode, Kerala were enrolled in this study. Pregnant patients, patients with diagnosed malignancy, and those with prior intestinal resection were excluded. 

Specific data collection forms were used. A detailed and proper history was taken including the SARC-F questionnaire with special emphasis on diet and nutrition. A thorough physical examination was done. Patients' height, weight, body mass index, hand grip using a dynamometer, and gait speed assessment by the four-meter walk test were assessed. Blood investigations like complete blood count, renal function test, liver function test, C-reactive protein (CRP), and erythrocyte sedimentation rate (ESR) were done. From clinical history, imaging, and endoscopic tests, the disease was characterized according to the Montreal classification. The CDAI (Crohn’s Disease Activity Index) score to assess disease severity was calculated. From colonoscopy, the SES-CD (Simple Endoscopic Score for Crohn’s Disease) was assessed to determine endoscopic severity at initial presentation.

Screening for sarcopenia was done using the SARC-F questionnaire. A hand grip dynamometer was used to assess hand grip strength, three consecutive values were assessed, and the highest value was taken. A grip strength of <27 kg in male patients and < 16 kg in female patients was considered probable sarcopenia. The skeletal muscle index was calculated using the L3 Skeletal muscle circumference from the CT scan abdomen image of the patient. CT scan abdomen is a part of the routine evaluation of CD to assess disease extent, identify perforation and intra-abdominal collections, and identify any fistulas and bowel wall thickening. No additional imaging was required to assess skeletal muscle volume. The cross-sectional area of skeletal muscles at the level of L3 vertebra was measured on CT. A cross-sectional image at the level of L3 vertebrae as a DICOM file was analyzed using the National Institute of Health Image J software, by keeping specific tissue demarcation between -29 to +150 Hounsfield units. The skeletal muscles at the L3 level include the psoas, erector spinae, quadratus lumborum, transversus abdominis, internal oblique, external oblique, and rectus abdominis. The L3 skeletal muscle index (SMI) which is the L3 skeletal muscle area divided by the height squared is used to assess the skeletal muscle volume. Sarcopenia is defined as SMI (Skeletal Muscle Index) < 36.5 cm²/m² and 30.2 cm²/m² for male patients and female patients, respectively. The gait speed measurement was done to grade sarcopenia, a four-meter walk test was done and speed was measured using a stopwatch. A speed < 0.8 m/sec was considered as severe sarcopenia. Patients were followed up for six months to assess achievement of remission and other clinical events. Patients were followed up during outpatient department visits. Patients were assessed for sarcopenia. 

Data was collected in a structured format and data was entered in Excel and analysis was done using IBM SPSS Statistics for Windows, Version 18 (Released 2009; IBM Corp., Armonk, New York, United States). Quantitative variables are expressed in mean and standard deviation. Qualitative variables were expressed in percentages. Statistical tests for quantitative variables were done using the chi-square test, and one quantitative analysis and one qualitative analysis were done using Student's t-test or ANOVA. All statistical tests were two-tailed, and a significance level (p-value) of 0.05 was used. 

## Results

A total of 70 newly diagnosed CD patients were enrolled in the study. Out of 70 patients, 26 (37.1%) were female patients and 44 (62.9%) were male patients. The mean age of the study population was 29.86 ± 9.896, with a minimum age of 18 and a maximum of 52. The mean height of the study population was 161.43 ± 7.262 cm. The mean weight was 47.67 ± 9.83 kilograms. The mean BMI was 18.20 ± 3.23 kg/m^2^ (Table 1).

**Table 1:** Baseline demographics and laboratory parameters of patients. BMI: Body mass index; CRP: C-reactive protein; ESR: Erythrocyte sedimentation rate.

Characteristics	Number
Mean age (In years)	29.86 ± 9.896
Females	26 (37.1 %)
Males	44 (62.9 %)
Height (in centimeter)	161.43 ± 7.262
Weight (in kilogram)	47.67 ± 9.83
BMI (kg/m^2^)	18.20 ± 3.23
Hemoglobin (g/dl)	10.15 ± 1.52
Albumin (g/dl)	3.23 ± .47
CRP (mg/L)	49.23 ± 40.87
ESR (mm/hour)	54.49 ± 19.84

Concerning disease behavior, the majority of the patients had B1 (nonstricturing/nonpenetrating) disease. A total of 38 patients had a history of perianal disease. The majority of the patients had ileocolonic (L3) involvement (Table 2). 

**Table 2:** Disease behavior and location - Montreal Classification.

Disease behaviour & location	Number Of Patients
B1 (nonstricturing, nonpenetrating)	41
B2 (stricturing)	24
B3 (penetrating)	4
B2+B3 (stricturing+ penetrating)	1
Perianal	38
L1 (ileal)	7
L2 (colonic)	3
L3 (ileocolonic)	50
L1+L4 (ileal+ Upper GI)	8
L3+L4 (ileocolonic+ Upper GI)	2

Clinical and endoscopic severity of patients were assessed at recruitment using CDAI (Crohn’s disease activity index scoring) and SES CD (simple endoscopic severity scores). At the first recruitment, the majority of patients had a CDAI score between 220 and 450 (Table 3). The mean CDAI score of the population was 251.47 ± 60.862. The mean SES CD score of the population was 9.89 ± 5.0.

**Table 3:** Clinical severity of the study group at diagnosis. CDAI: Crohn's disease activity index.

CDAI	Number of patients
<150	0
150- 220	26
220-450	44
>450	0

On the SARC-F questionnaire, 33 (47.1%) patients had a score more than equal to 4. All the patients were subjected to hand grip assessment with a hand-held dynamometer and the L3 skeletal muscle Index was calculated with Image J software using a DICOM image from a cross-sectional imaging CT scan at the L3 level. The mean hand grip (kg) in the dominant hand of the study population was 30.991 ± 13.08 kg and the mean L3 skeletal muscle index was 37.35 ± 6.63 cm^2^/m^2^. 

The prevalence of sarcopenia in this study according to the L3SMI was 38.6% (27 patients). Sarcopenia was confirmed by L3SMI values < 36.5 cm²/m² and 30.2 cm²/m² for male patients and female patients, respectively. A four-meter walk test was done in all patients. Fifteen patients with sarcopenia confirmed on L3SMI couldn’t complete four meters at a speed of 0.8 m/s, labeled as severe sarcopenia; 55% of patients with sarcopenia had severe sarcopenia according to a walk test.

Patients were divided into two groups, with sarcopenia and without sarcopenia and were assessed for disease severity using the CDAI score. Sarcopenia was more prevalent in patients with higher clinical severity. Patients with sarcopenia had a higher CDAI score at presentation, and the difference in the CDAI between the two groups was statistically significant (p-value <0.001) (Table 4). The mean SES CD score in the sarcopenia group was 11.22 ± 5.04 and in the group without sarcopenia was 7.68 ± 3.60. 

**Table 4:** CDAI score - Clinical disease severity and sarcopenia. CDAI: Crohn's Disease Activity Index. Statistical significance was determined using the chi-square test. A p-value of <0.05 indicates statistical significance.

CDAI Score At 1^st^ Month	No Sarcopenia	Sarcopenia	Test Statistic (chi-square)	p-value
150-220 (Mild-Moderate)	55.81% (24)	7.40% (2)	16.646	<0.001
220-450 (Moderate-Severe)	44.1% (19)	92.6 % (25)		

In this study, the mean age in the sarcopenia group was 24.52 ± 8.225 years and in those without sarcopenia, it was 33.21 ± 9.443. It was seen that in those with a younger age of onset of CD, sarcopenia was more prevalent. The difference in age between the two groups was statistically significant (t= -3.934, p <0.001). Concerning the prevalence of sarcopenia according to gender, it was noted that there was no statistically significant difference between male patients and female patients (p-value= 0.316) (Table 5).

**Table 5:** Sarcopenia and correlation with gender. Statistical significance was determined using the chi-square test.

Variable	No Sarcopenia	Sarcopenia	Test statistic (chi-square)	p-value
Male	67.4 % (29)	55.6 % (15)	1.004	0.316
Female	32.6 % (14)	44.4 % (12)		

In our study, the mean height was not statistically different in the two groups (p-value =0.217). The mean weight and BMI were lower in the sarcopenia group and it was found to be statistically significant (p-value <0.001). Mean handgrip strength in the dominant hand in the sarcopenia group was 19.00 ± 6.48 kg which was lower when compared to those without sarcopenia and this was statistically significant (p-value <0.001). The mean L3 skeletal muscle area and L3 skeletal muscle index was lower in the sarcopenia group and it was statistically significant (p-value <0.001) (Table 6).

**Table 6:** Sarcopenia - correlation with height, weight, BMI, hand grip strength, mean L3 skeletal muscle area, and mean skeletal muscle index. cm: Centimeter; kg: Kilogram. Statistical significance was determined using an independent t-test. A p-value of <0.05 indicates statistical significance.

Variable	No Sarcopenia	Sarcopenia	Test statistic (t)	p-value
Height (in cm)	162.37 ± 5.835	159.93 ± 9.00	-1.255	0.217
Weight (in kg)	53.05 ± 7.64	39.11 ± 6.18	-7.969	<0.001
BMI (in kg/m^2^)	20.13 ± 2.47	15.12 ± 1.33	-10.980	<0.001
Hand grip strength dominant hand (in kg)	38.52 ± 10.24	19.00 ± 6.48	-8.840	<0.001
L3 skeletal muscle area cm^2 ^(mean)	110.01 ± 13.78	78.01 ± 13.44	-9.540	<0.001
L3 skeletal muscle index cm^2^/m^2 ^(mean)	41.71 ± 3.68	30.41 ± 3.65	-12.528	<0.001

The mean hemoglobin at the one-month follow-up and after a six-month follow-up was lower in the sarcopenia group when compared to patients in the no-sarcopenia group and this was found to be statistically significant (p-value <0.001). Mean albumin in the sarcopenia group at the one-month follow-up and after a six-month follow-up was lower when compared to those in the no sarcopenia group and this was found to be statistically significant (p-value <0.001) (Table 7).

**Table 7:** Sarcopenia correlation with hemoglobin and albumin in both groups at one- and six-month follow-up. Statistical significance was determined using an independent t-test. A p-value of <0.05 indicates statistical significance.

	Hemoglobin (g/dl)		Albumin (g/dl)	
	1^st^ Month	6^th^ Month	1^st^ month	6^th^ month
No sarcopenia	10.85 ± 1.39	11.09 ±1.40	3.41 ± .46	3.54 ± .36
Sarcopenia	9.03 ± .95	9.61 ± 1.05	2.94 ±.31	3.17 ± .23
Test statistic (t)	-5.923	-4.679	-5.007	-5.157
p=value	<0.001	<0.001	<0.001	<0.001

The mean CRP and ESR values were higher in the sarcopenia group at one month and at the end of the six-month follow-up when compared to no sarcopenia group and this was statistically significant (Table 8).

**Table 8:** Sarcopenia correlation with CRP and ESR at one and six-month follow-up. CRP: C-reactive protein; ESR: Erythrocyte sedimentation rate. Statistical significance was determined using an independent t-test. A p-value of <0.05 indicates statistical significance.

	CRP (mg/l)		ESR (mm/hour)	
	1^st^ Month	6^th^ Month	1^st^ month	6^th^ month
No Sarcopenia	39.40 ± 36.60	14.53 ± 12.02	48.44 ± 18.82	31.19 ± 15.71
Sarcopenia	64.88 ± 43.08	28.62 ± 25.96	64.11 ± 17.79	38.85 ± 15.13
Test statistic (t)	2.647	2.649	3.461	2.015
p-value	0.010	0.012	0.001	0.048

The mean CDAI score at the one-month follow-up in the no sarcopenia group was 222.44 ± 40.258 and in the sarcopenia group, it was 297.70 ± 59.99. Clinical disease severity assessed by CDAI scoring was higher in the sarcopenia group at diagnosis when compared to no sarcopenia group and this was statistically significant (p-value <0.001). The mean CDAI score at the six-month follow-up in the no sarcopenia group was 112.33 ± 34.26 and in the sarcopenia group, it was 176.85 ± 79.76. Clinical disease severity at the six-month follow-up was higher in the sarcopenia group and this was statistically significant (p-value <0.001) (Table 9).

**Table 9:** Clinical severity at one and six-month follow-up using the CDAI score. CDAI: Crohn's Disease Activity Index. Statistical significance was determined using an independent t-test. A p-value <0.05 indicates statistical significance.

	CDAI at 1^st^ Month	CDAI at 6^th^ Month
No sarcopenia	222.44 ± 40.258	112.33 ± 34.26
Sarcopenia	297.70 ± 59.99	176.85 ± 79.76
Test statistic (t)	6.287	3.979
p-value	<0.001	<0.001

At the six-month follow-up, 81.4% of patients in the no sarcopenia group were in clinical remission with a CDAI score <150, whereas only 48.1% of those in the sarcopenia group were in remission. The sarcopenia group had a higher clinical disease severity at the six-month follow-up and also decreased clinical disease remission and this was found to be statistically significant (chi-square=8.507, p-value =0.007). The response to treatment was assessed by a fall in the CDAI score of >100 at six-month follow up, and it was noted that 66.7% of patients had response in the sarcopenia group which was lower when compared to the group without sarcopenia who had a response rate of 88.4% and it was statistically significant (chi-square=4.884, p-value=0.027). The mean number of flares was noted to be 1.07 ± 1.035 in the sarcopenia group which was higher when compared to those without sarcopenia in whom it was .63 ± .691 and this was statistically significant (t=2.165, p-value=0.034). 

## Discussion

This prospective cohort study to assess the prevalence of sarcopenia and its correlation with disease activity and effect on prognosis included 70 patients with newly diagnosed IBD, i.e., CD. The prevalence of sarcopenia in our study was 38.6% (n=27). In a previous retrospective study conducted by Lee et al. [18] in Seoul, the prevalence of sarcopenia was 51%. The study was done using the L3 skeletal muscle index as the indicator of sarcopenia. Another retrospective study conducted by Boparai et al. [17] at AIIMS New Delhi in 44 patients had a 43% prevalence of sarcopenia in CD. They also used the L3 skeletal muscle index. Our study showed that there is an increased prevalence of sarcopenia in CD patients. 

The mean age of our study population was 29.86 ± 9.896 years. In a study done by Boparai et al. [18], the mean age of onset was 34.14 ± 14. In the study done by Lee et al. [18], the mean age was 29 years. In our study, 37.1% (n=26) were female patients and 62.5% (n=44) were male patients. The mean height of the population in our study group was 161.43 ± 7.262 cm, and the mean body weight was 47.67 ± 9.83 kg. The mean BMI was 18.20 ± 3.23 kg/m^2^. The BMI in the no sarcopenia group was 20.13±2.47kg/m^2^ and in the sarcopenia group, it was 15.12 ± 1.33 kg/m^2^. BMI was lower in the sarcopenia group and was statistically significant. Boparai et al. [17] also found that BMI was lower in the sarcopenia group.

The mean L3 skeletal muscle index (L3SMI) of our population was 37.35 ± 6.63 cm^2^/m^2^. The mean L3 skeletal muscle index of patients with sarcopenia was 30.41 ± 3.65 cm^2^/m^2^ and without sarcopenia, it was 41.71 ± 3.68 cm^2^/m^2^. This showed a statistically significant difference in the two groups. In a previous Indian study by Boparai et al. [17], the mean L3SMI was 27.45 ± 4.9 cm^2^/m^2^ in the sarcopenia group and 39.55 ± 4.89 in the no sarcopenia group. Our study showed no statistically significant difference between males and females in the prevalence of sarcopenia. Mean hemoglobin in the sarcopenia group was 9.03 ± 0.95 g/dl at one-month and 9.61 ± 1.05 g/dl at six-month follow-up. The hemoglobin level was lower in the sarcopenia group. Hemoglobin showed a statistically significant difference between the two groups. This shows that sarcopenia correlated with the degree of anemia. The mean albumin was 3.41 ± 0.46 g/dl at 1-month and 3.54 ± 0.36 g/dl at six-month follow-up in the no sarcopenia group and 2.94 ± 0.31 g/dl at one month and 3.17 ± 0.23 g/dl at six-month follow-up in the sarcopenia group. Serum albumin levels showed a difference in statistical significance between the two groups. A previous study by Lee et al. [18] showed a statistically significant difference in hemoglobin and albumin among both groups but could not demonstrate any prognostic significance. 

Concerning CRP and ESR, this study showed that there was a statistically significant difference in CRP levels between the two groups with and without sarcopenia at one-month and six-month follow-up; levels were higher in patients in the sarcopenia group. ESR at one-month and six-month follow-ups showed a statistically significant difference between the two groups and was higher in the sarcopenia group. Lee et al. [18] showed that CRP levels were higher in the sarcopenia group, and ESR correlated with sarcopenia. This shows that inflammation plays an important role in causing sarcopenia. 

Regarding the baseline CDAI score at diagnosis, our study had higher CDAI scores at first presentation in the sarcopenia group, clinical disease severity was higher in the sarcopenia group, and this showed statistical significance. The mean endoscopic severity score assessed by SES CD score was 7.68 ± 3.60 in the no sarcopenia group and 11.22 ± 5.04 in the sarcopenia group. Clinical and endoscopic disease severity was higher in the sarcopenia group in our study. 

There is robust evidence to show that sarcopenia has a detrimental effect on patients' physiological reserve and their ability to recover postoperatively [19]. Recent studies show that IBD is associated with an increased risk of sarcopenia, which impacts the clinical course and response to therapy [20]. In our study, the response to therapy was lower in the sarcopenia group at the six-month follow-up, and this was statistically significant. Disease flares were also more common in the sarcopenia group. 

Our study is not without its limitations. One of the limitations of this study was that it was a single-center study. The follow-up period was short, and hence mortality and survival assessments could not be assessed. Endoscopic severity assessment was not done at six-month follow-up. 

## Conclusions

There are limited studies regarding the prevalence of sarcopenia in CD patients and its prognostic significance in CD. There is no data available from Kerala, a southern state in India regarding the prevalence of sarcopenia in CD patients and its effect on disease activity, and prognosis. The results from our study show that sarcopenia is prevalent in CD patients in the Northern Kerala population. Sarcopenia prevalence is higher in CD patients with higher clinical and endoscopic severity. Patients with sarcopenia had higher levels of CRP and ESR at diagnosis and six-month follow-up. Prognosis assessment at six-month follow-up showed reduced remission rates and lower response to treatment in patients who had sarcopenia. 

Going by our study results, it would be ideal that all patients with newly diagnosed CD be screened for sarcopenia by using hand grip strength and L3SMI, as it was shown that sarcopenia is an important consideration in the management of IBD, in both prognostication and treatment. Early detection and management of sarcopenia may help in improving treatment outcomes. 
